# Editorial: Langerhans Cells and How Skin Pathology Reshapes the Local Immune Environment

**DOI:** 10.3389/fimmu.2019.00139

**Published:** 2019-02-07

**Authors:** Clare L. Bennett, Carrie A. Ambler

**Affiliations:** ^1^Institute for Immunity and Transplantation University College London, London, United Kingdom; ^2^Biosciences Department, Durham University, Durham, United Kingdom

**Keywords:** langherans cells, skin, graft vs. host disease, psoriasis, pathology

The skin is an important barrier site. It contains a dynamic network of immune cells that ensure tolerance to daily insults, whilst maintaining the capacity to rapidly initiate innate and adaptive immune responses to more threatening challenges. In the steady state, the biology of the development and function of most skin immune cells has been well-defined. However, immune pathology, driven by infection or autoimmune triggers, may extensively damage both the local architecture, and alter the immune cell compartment. Little is known about how these acute changes reshape the tissue environment or immune homeostasis once pathology has resolved. One example is cutaneous graft-vs.-host disease (GVHD) that occurs when donor T cells attack healthy skin cells after allogeneic hematopoietic stem cell transplant (allo-HSCT). Here, T cell pathology not only dramatically alters the local inflammatory environment, but destruction of resident cells by cytotoxic T cells may lead to re-organization of the immune cell network. However, the long-term consequences of these changes for immune function in the skin are unknown.

Langerhans cells (LC) are antigen presenting cells which are unique to the skin epidermis, although similar cells may be found at other epithelial sites, such as the oral mucosa. LC were first identified by Paul Langerhans in the nineteenth Century, due to their striking dendritic morphology in the skin epidermis, but it took another 100 years before their importance in skin immunity began to be appreciated by early pioneers in the field [for a comprehensive review see (1)]. More recently, shifts in our understanding of the developmental biology of LC have lead researchers to describe new functions for LC in the skin and draining lymph nodes (LN).

This research topic brings together six articles that provide both insight into our current understanding of LC and in health and disease, and new perspectives in our view of LC function. Further, we have included a seventh article, by Li et al. in the format of a methods paper, presented to quantify and measure some of the skin's immunological responses. The field is heavily dependent on assays, such as caliper-based measurements of wound size, tumor growth, and ear swelling that are subject to user variability, and here we present novel, unbiased approaches for quantification.

Deckers et al. present our current understanding of the ontogeny and function of LC, and their involvement in skin disease, with a focus on allergy and autoimmunity. Following this, a series of reviews present novel concepts in current thinking on how LC function, and are wired. Sagi and Heironymus explore the concept that migration of LC out of the skin to LN requires activation of an epithelial to mesenchymal transition (EMT), which is more commonly associated with the acquisition of metastatic and migratory potential of carcinomas. They discuss current knowledge of Met/HBFG signaling in LC and dendritic cells (DC), and propose the importance of this signaling pathway for LC mobilzation and therefore function. This is complimented by the mini-review by West and Bennett who explore the concept that our historical focus on LC as cells that pre-dominantly migrate to LN to prime T cell immunity, may have masked our appreciation of other roles directly in the skin. They argue that LC function *in situ* within the epidermis is consistent with their embryonic origin as tissue macrophages. Finally, Clayton et al. have utilized novel transcriptomics approaches to define gene regulatory networks in human LC, and assess how these may provide clues to LC development and function. This work highlights the uniqueness of epidermal LC compared to macrophages and DC.

In two subsequent reviews, we focus on the role of LC in clinical disease. In their mini-review, Santos e Sousa et al. discuss the pathology associated with clinical graft-vs.-host disease (GVHD), and how this alters the skin immune environment. LC are radio-resistant, and persist as the major allogeneic professional antigen presenting cell population in the skin of patients after allo-HSCT. This, with the assumption of their dominant role in priming T cell immunity, led to the long-held view that they were responsible for cutaneous GVHD. However, recent work has challenged this view, and the role of LC in GVHD pathology remains controversial. Santos e Sousa et al. present their novel finding that epidermal LC drive GVHD by directly regulating T cell function in the skin. In comparison, Eidsmo and Martini discuss the direct role of LC for on-going pathology during psoriasis. Disease is characterized by the recurrence of lesions at fixed sites. Here, they discuss evidence that LC may maintain a transcriptional “memory” of pathology, perhaps providing a biological explanation for the localization of the pathological response.

Fundamentally, this research topic represents our growing understand of the varied roles LCs play and how they contribute to skin immunity in the steady state and in disease.

## Author Contributions

All authors listed have made a substantial, direct and intellectual contribution to the work, and approved it for publication.

### Conflict of Interest Statement

The authors declare that the research was conducted in the absence of any commercial or financial relationships that could be construed as a potential conflict of interest.

## References

[1] Romani N Brunner P Stingl G. Changing views of the role of Langerhans cells. J Invest Dermatol. (2012) 132:872–81. 10.1038/jid.2011.437 22217741

